# Supplementary figures and images for: Lower rotational inertia and larger leg muscles indicate more rapid turns in tyrannosaurids than in other large theropods
Source: PeerJ. 2019 Feb 21;7:e6432. doi: 10.7717/peerj.6432 (PMC6387760; doi:10.7717/peerj.6432)

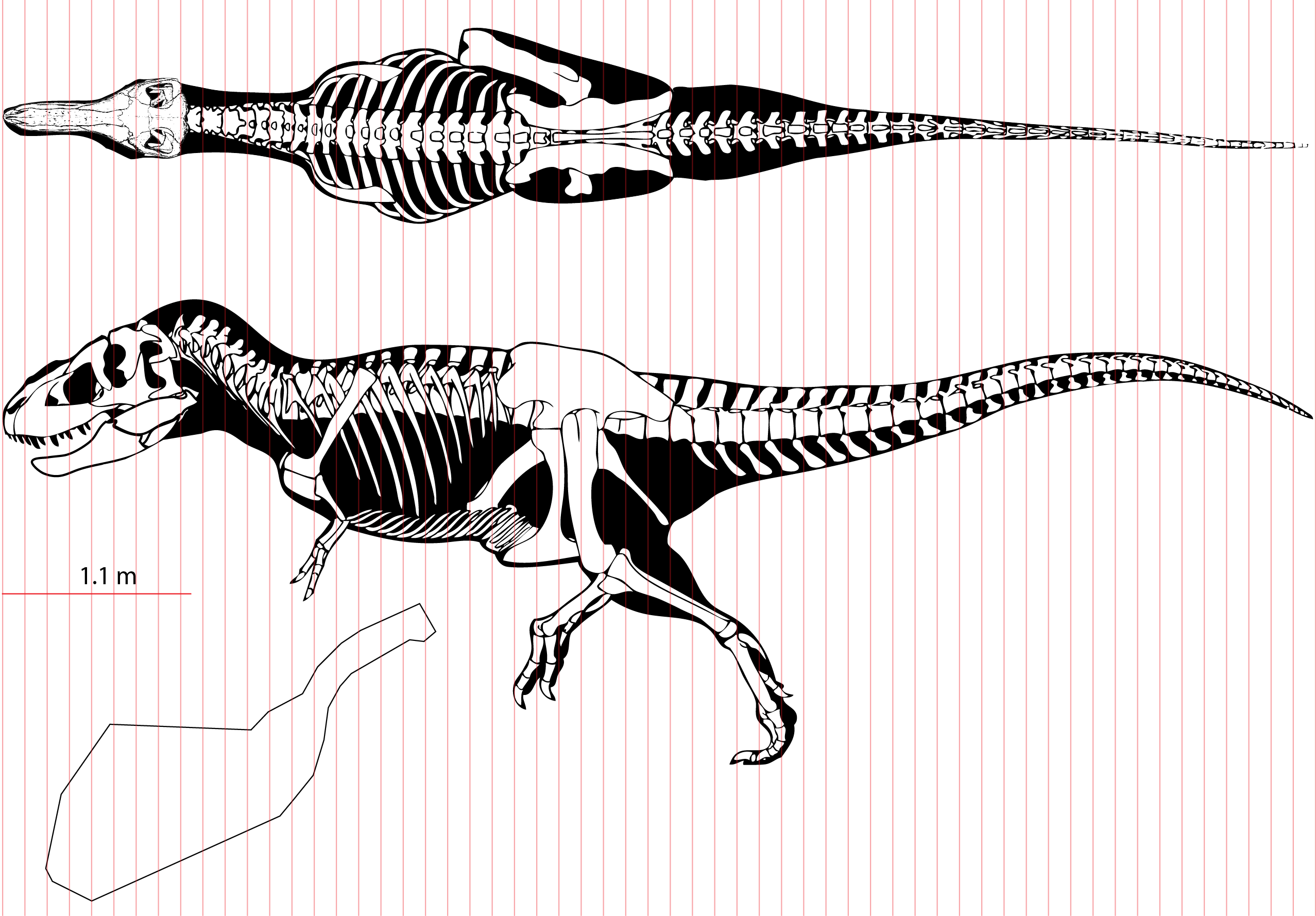

Supplement: Supplemental Information 2 — This image was used for digitizing outlines and calculating mass properties of Tarbosaurus bataar (ZPAL MgD I/4). The skull is tilted down in lateral view, and shortened in dorsal view to match the length. See text for details. The tail is restores as moderately wide, after Persons & Currie (2011a). [file peerj-07-6432-s002.png]

## A. Agility Force Planted

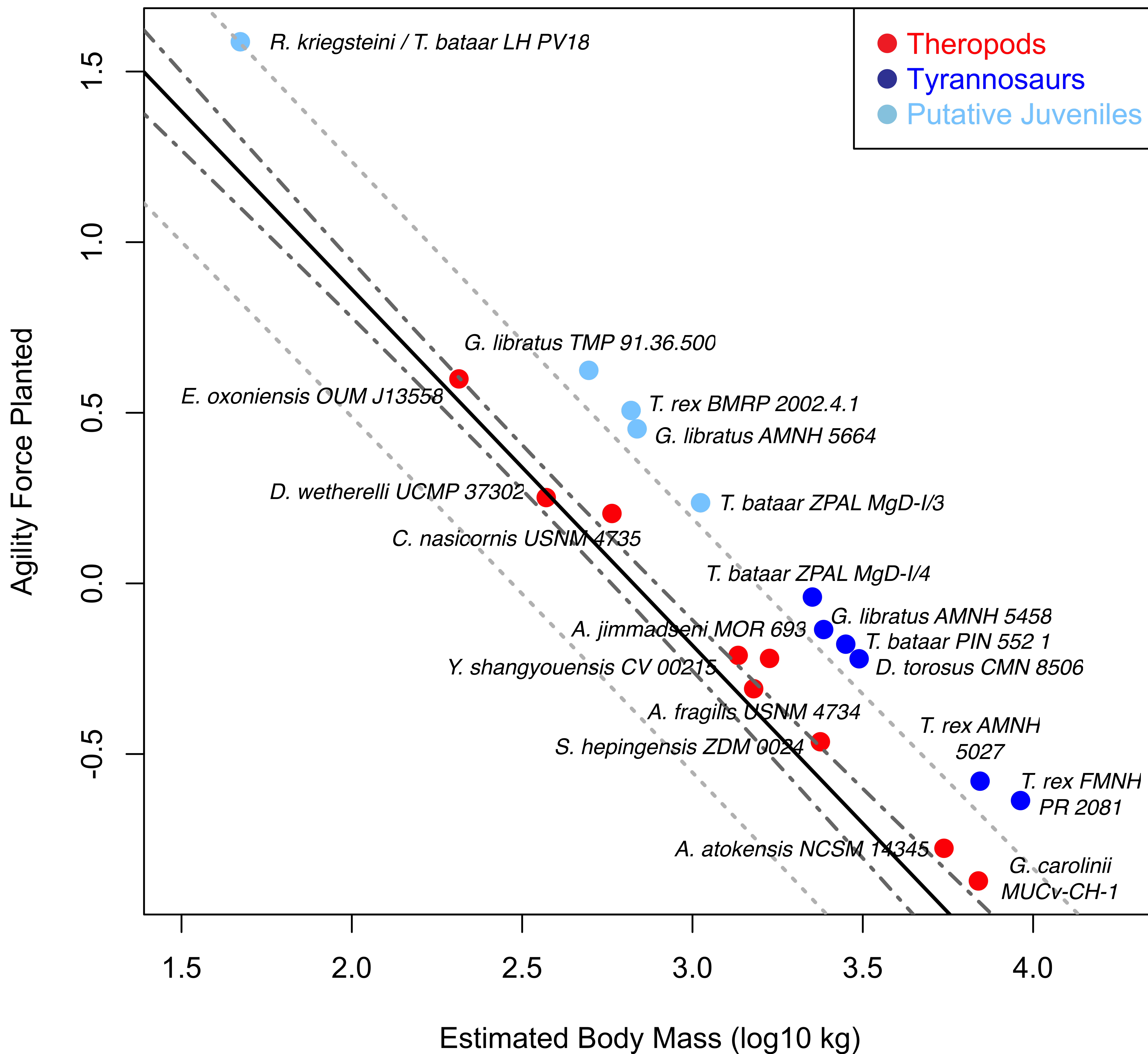

## B. Agility Moment Planted

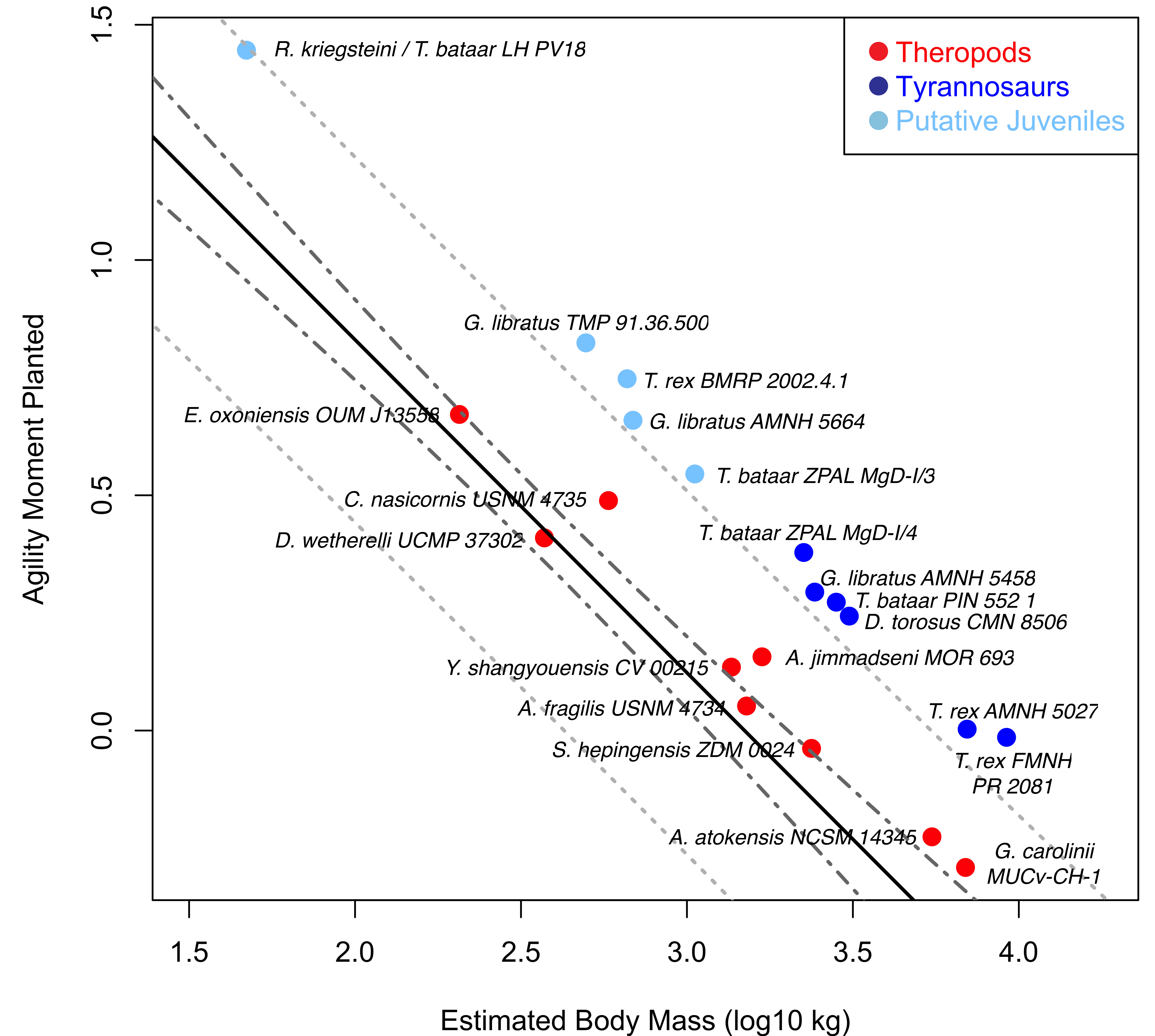

Supplement: Supplemental Information 6 — This figure is the same as text Fig. 4, but with full labels for all data points including taxa, and juvenile or adult status and specimen numbers for multi-specimen taxa. [file peerj-07-6432-s006.pdf]

## A. Agility Force en Pointe

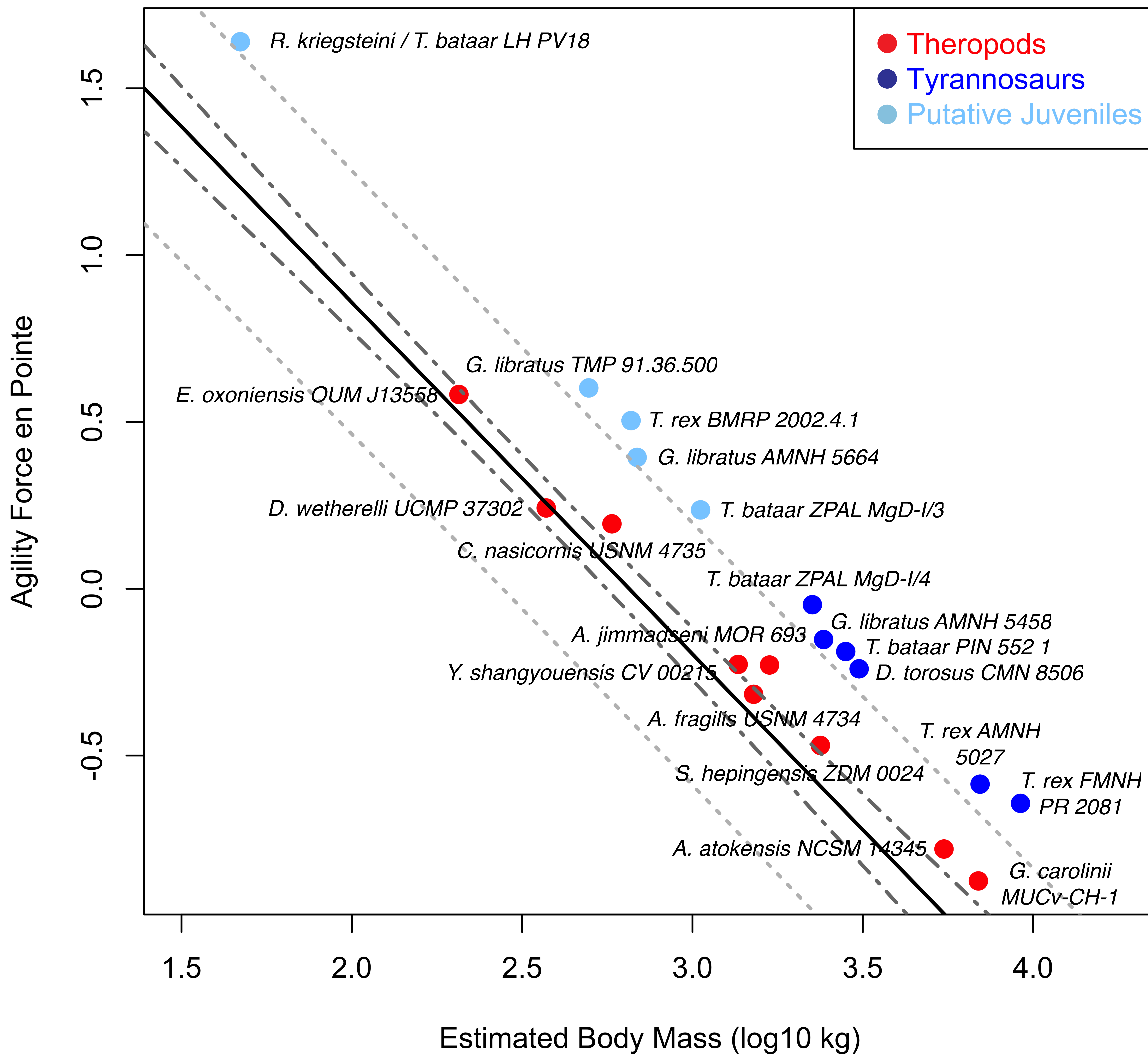

## B. Agility Moment en Pointe

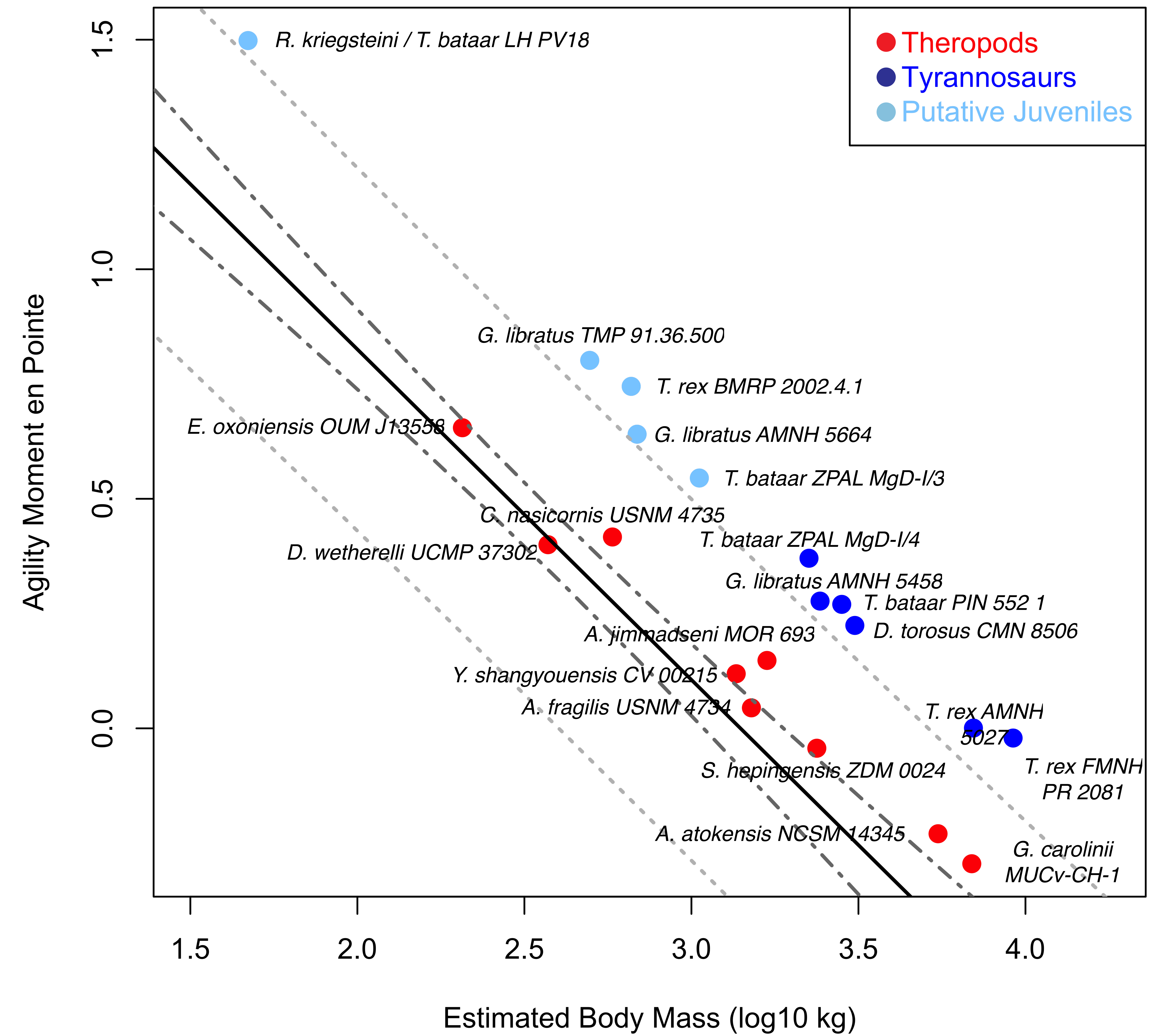

Supplement: Supplemental Information 7 — This figure is the same as text Fig. 5, but with full labels for all data points including taxa, and juvenile or adult status and specimen numbers for multi-specimen taxa. [file peerj-07-6432-s007.pdf]
